# NCP-BiRW: A Hybrid Approach for Predicting Long Noncoding RNA-Disease Associations by Network Consistency Projection and Bi-Random Walk

**DOI:** 10.3389/fgene.2022.862272

**Published:** 2022-04-13

**Authors:** Yanling Liu, Hong Yang, Chu Zheng, Ke Wang, Jingjing Yan, Hongyan Cao, Yanbo Zhang

**Affiliations:** ^1^ Department of Health Statistics, School of Public Health, Shanxi Medical University, Taiyuan, China; ^2^ Department of Mathematics, Changzhi Medical College, Changzhi, China; ^3^ Shanxi Provincial Key Laboratory of Major Diseases Risk Assessment, Taiyuan, China; ^4^ School of Health and Service Management, Shanxi University of Chinese Medicine, Taiyuan, China

**Keywords:** lncRNA-disease association prediction, integrated similarity, network consistency projection, normalization, bi-random walk

## Abstract

Long non-coding RNAs (lncRNAs) play significant roles in the disease process. Understanding the pathological mechanisms of lncRNAs during the course of various diseases will help clinicians prevent and treat diseases. With the emergence of high-throughput techniques, many biological experiments have been developed to study lncRNA-disease associations. Because experimental methods are costly, slow, and laborious, a growing number of computational models have emerged. Here, we present a new approach using network consistency projection and bi-random walk (NCP-BiRW) to infer hidden lncRNA-disease associations. First, integrated similarity networks for lncRNAs and diseases were constructed by merging similarity information. Subsequently, network consistency projection was applied to calculate space projection scores for lncRNAs and diseases, which were then introduced into a bi-random walk method for association prediction. To test model performance, we employed 5- and 10-fold cross-validation, with the area under the receiver operating characteristic curve as the evaluation indicator. The computational results showed that our method outperformed the other five advanced algorithms. In addition, the novel method was applied to another dataset in the Mammalian ncRNA-Disease Repository (MNDR) database and showed excellent performance. Finally, case studies were carried out on atherosclerosis and leukemia to confirm the effectiveness of our method in practice. In conclusion, we could infer lncRNA-disease associations using the NCP-BiRW model, which may benefit biomedical studies in the future.

## Introduction

Long non-coding RNAs (lncRNAs) were primitively considered noise in transcriptional regulation and thought to have no biological functions (Guttman et al., 2013; Li et al., 2019). In recent decades, however, lncRNAs have attracted growing attention from researchers worldwide owing to the discovery of their critical biological functions. Increasing numbers of lncRNAs have been identified in eukaryotes (Guttman et al., 2009) and abnormal lncRNA expression has been shown to cause many human diseases, including nervous system diseases (Qureshi and Mehler, 2013; Chen et al., 2021), cardiovascular diseases (Bhatti et al., 2021; Xie et al., 2021), various cancers (Amelio et al., 2021; Taniue and Akimitsu, 2021), autoimmune diseases (Lodde et al., 2020; Zeni and Mraz, 2021), and blood diseases (Wei et al., 2013; Kirtonia et al., 2021). Therefore, searching for possible lncRNA-disease associations may facilitate the elucidation of the molecular pathogenesis of human diseases and could be relevant in disease diagnosis, prognosis, prevention, and treatment in the clinical setting. At present, researchers mainly study potential lncRNA-disease associations through biological experiment verification and computational model prediction. However, biological experiments are often costly, time-consuming, and inconclusive (Chen et al., 2017). Thus, few lncRNA-disease associations have been verified experimentally, and the use of more advanced algorithms is essential.

LncRNA-disease association predictive models can be roughly classified into two types, the first of which is machine learning-based. Chen and Yan (2013) proposed the calculative model LRLSLDA, which integrates known lncRNA-disease interactions and lncRNA expression profiles and applies the Laplacian regularized least square method to predict disease-related lncRNAs. Subsequently, Chen et al. (2015) developed LRLSLDA-LNCSIM. Under the hypothesis that lncRNAs with similar functions tend to be related to similar diseases, two new functional similarity computational models, LNCSIM1 and LNCSIM2, were developed. Then, the two models were combined with the LRLSLDA model for the prediction of lncRNA-disease associations. Yang et al. (2014) constructed a binary network for genes and diseases, and applied a network propagation algorithm to find hidden lncRNA-disease interactions. On the basis of the naïve Bayesian classifier, Zhao et al. (2015) developed a novel method to identify cancer-related lncRNAs by integrating genome, transcriptome, and regulome data and identified 707 lncRNAs. Furthermore, Lu et al. (2018) proposed SIMCLDA, which first computed disease functional similarity and lncRNA Gaussian interaction profile kernel similarity and then used principal component analysis to extract the principal eigenvector of disease and lncRNA similarity. Finally, the inductive matrix completion technique was used for association prediction. In recent years, there have been many deep learning techniques developed in the field of bioinformatics. Zeng et al. (2020) developed the SDLDA model to predict lncRNA-disease interactions. SDLDA extracted the features of lncRNAs and diseases, including the linear features acquired by the singular value decomposition technique and the non-linear features obtained by the deep learning method. Zeng et al. (2021) proposed a deep matrix factorization model called DMFLDA. Based on the lncRNA-disease associations matrix, the non-linear hidden layers of DMFLDA were employed to learn the latent representation of lncRNAs and diseases, which could capture more complex and nonlinear lncRNA-disease associations. However, negative samples are required for these machine learning methods and are difficult to obtain.

The second type of predictive model is network-based. Sun et al. (2014) constructed the RWRlncD model, in which random walk with restart was used to compute lncRNA functional similarity, and the lncRNA functional similarity network was then combined with the lncRNA-disease and disease similarity networks to form a global network. Finally, the candidate lncRNAs of specific diseases of interest were sorted. Chen (2015) developed KATZLDA, which integrated lncRNA functional similarity, lncRNA expression profiles, disease semantic similarity, Gaussian interaction profile kernel similarity, and the known lncRNA-disease pairs, and then used the KATZ method to predict the potential lncRNA-disease interactions. Wen et al. (2018) developed Lap-BiRWRHLDA. First, Laplacian normalization was applied to compute lncRNA similarity matrix and disease similarity matrix. Then a heterogeneous network was constructed based on lncRNA similarity network, disease similarity network, and known lncRNA-disease associations. Finally, bi-random walk algorithm was applied on this heterogeneous network to predict lncRNA-disease associations. Hu et al. (2019) proposed the BiWalkLDA model, which applied bi-random walk method to predict hidden lncRNA-disease associations. It integrated gene ontology and interaction profiles to calculate disease similarity, and used interaction profiles data to calculate lncRNA similarity in which the cold-start problem was solved by using the local topological structure of a new lncRNA. Xie et al. (2019) proposed NCPHLDA, which calculated the comprehensive similarity for lncRNAs and diseases and then applied a network consistency projection method to infer the interactions between lncRNAs and diseases. The most significant advantage of the network consistency projection algorithm is that it has no parameters. The network consistency projection algorithm and the bi-random walk algorithm have the common characteristic that they both have the calculation process on the similarity networks of lncRNAs and diseases. Wang and Yan (2019) constructed the IDLDA model, which used an improved diffusion method to infer lncRNA-disease interactions based on a combined dataset. Recently, some hybrid computational models have emerged and showed good performance. Xie et al. (2021) designed the RWSF-BLP model to forecast lncRNA-disease interactions. The model first applied a random walk algorithm to fuse various similarity networks and then adopted bidirectional label propagation to make predictions. Yin et al. (2020) created the NCPLP model based on network consistency projection and label propagation to predict microbe-disease interactions. These biological network-based methods provide a fresh perspective and framework with which we can construct new computational models.

Here, we intend to construct a hybrid method consisting of two different methods. According to previous studies, we considered the following three factors in modeling: First, the two methods could be combined properly and reasonably. Second, it is better to have no more parameters, which is directly related to computational efficiency. Third, the combination of two methods should contribute more biological information to the final result. Accordingly, in this paper, we come up with a hybrid method consisting of network consistency projection and bi-random walk (NCP-BiRW) to infer lncRNA-disease interactions. We investigated comprehensive similarity networks for lncRNAs and diseases based on known lncRNA-disease relationships, disease semantic similarity, lncRNA functional similarity, and Gaussian interaction profile (GIP) kernel similarity for lncRNAs and diseases to apply more similarity information. Second, we constructed a heterogeneous network consisting of lncRNA similarity network, disease similarity network, and lncRNA-disease association network. The network consistency projection method was used to compute lncRNA network projection scores and disease network projection scores. Third, we added the results of the network consistency projection algorithm to the bi-random walk algorithm, and finally got the predicted scores of potential lncRNA-disease interactions. Five- and ten-fold cross-validation (CV) were adopted to verify the effectiveness of NCP-BiRW. Our Results demonstrated that our method outperformed the other five classical algorithms and we showed that the model was robust when applied to another dataset. Finally, case studies on atherosclerosis and leukemia were used to further verify the validity of our model.

## Materials and Methods

### Long non-coding RNA-Disease Associations Dataset

We downloaded known lncRNA-disease associations from the 2017-version LncRNADisease database (Chen et al., 2013) (http://www.cuilab.cn/lncrnadisease). After conducting data quality control and data cleaning, 701 known experimentally validated interactions between 157 diseases and 82 lncRNAs were acquired, as previously reported (Fan et al., 2020). $n_{l}$ and $n_{d}$ indicate the numbers of lncRNAs and diseases, respectively. $A={\left\{A_{ij}\right\}}_{n_{l}\times n_{d}}$ denotes the association matrix, where $A_{ij}$ is defined as follows:

$$A_{ij}=\{\begin{array}{cc} 1 & \ln cRNA\;l_{i}\;is\;associated\;with\;disease\;d_{j}\\ & 0\;\;\;\;\;\;\;\;\;\;\;\;\;\;\;\;\;\;\;\;\;\;\;\;\;\;\;\;\;otherwise \end{array}$$ (1)

### Gaussian Interaction Profile Kernel Similarity for Long non-coding RNAs and Diseases

Researchers have hypothesized that the more similar two lncRNAs are, the more likely they are to have similar interaction modes with similar diseases (van Laarhoven et al., 2011). Thus, GIP kernel similarity was used to measure the similarities of lncRNAs and diseases. Given lncRNA $l_{i}$ and lncRNA $l_{j}$ , the GIP kernel similarity between the two lncRNAs can be calculated as follows:

$$KL\left(l_{i}\;,l_{j}\right)=\exp\left(-\gamma_{l}{\left\|IP\left(l_{i}\right)-IP\left(l_{j}\right)\right\|}^{2}\right)$$ (2)

$$\gamma_{l}=\frac{\gamma_{l}^{\prime }}{\left(\frac{1}{n_{l}\;}\sum_{i=1}^{n_{l}}{\left\|IP\left(l_{i}\right)\right\|}^{2}\right)}$$ (3)

where KL represents the GIP kernel similarity matrix of lncRNAs, $IP\left(l_{i}\right)$ indicates the *i*-th row of A , $\gamma_{l}$ is the normalized kernel bandwidth, and $\gamma_{l}^{\prime }$ is a parameter that is often set as 1 (van Laarhoven et al., 2011).

Similarly, the GIP kernel similarity of disease is calculated as follows:

$$KD\left(d_{i},d_{j}\right)=exp\left(-\gamma_{d}{\left\|IP\left(d_{i}\right)-IP\left(d_{j}\right)\right\|}^{2}\right)$$ (4)

$$\gamma_{d}=\frac{\gamma_{d}^{\prime }}{\left(\frac{1}{n_{d}\;}\sum_{i=1}^{n_{d}\;}{\left\|IP\left(d_{i}\right)\right\|}^{2}\right)}$$ (5)

where KD represents the GIP kernel similarity matrix of diseases, $IP\left(d_{i}\right)$ denotes the *i*-th column of A , $\gamma_{d}$ indicates the normalized kernel bandwidth, and $\gamma_{d}^{\prime }=1$ .

### Disease Semantic Similarity

Directed acyclic graphs (DAGs) have been widely used to compute the semantic similarity between diseases when predicting potential lncRNA-disease interactions (Chen et al., 2017). Here, the disease semantic similarity was calculated as previously reported (Fan et al., 2020). First, the Medical Subject Headings (MeSH) descriptors of the diseases we needed were downloaded from the National Library of Medicine (http://www.nlm.nih.gov/) (Wang et al., 2010). We then constructed a DAG for each disease d : DAG(d)=(d,N(d),E(d)) , where N(d) represents all the ancestor nodes of d (containing d ), and E(d) denotes all the direct edges from parent nodes to child nodes. For a disease *s* in DAG(d) , its semantic contribution to disease d is computed as follows:

$$D_{d}\left(s\right)=\{\begin{array}{cc} 1 & if\;s=d\\ \max\left\{\left(\Delta +P_{s}\right)\times D_{d}\left(s\prime \right)|s\prime \in children\;of\;s\right\} & if\;s\neq d \end{array}$$ (6)

where Δ denotes the semantic contribution factor and is set to 0.5 (Wang et al., 2010). $P_{s}$ is defined as:

$$P_{s}=\frac{\underset{k\in K}{\max}\left\{dags\left(k\right)\right\}-dags\left(s\right)}{D}$$ (7)

where *K* is the diseases set in MeSH, dags(s) is the number of DAGs containing *s,* and D represents the number of all diseases in MeSH.

By accumulating the semantic contributions of all the diseases in DAG(d) , the following formula is used to compute the final semantic similarity of disease d :

$$DV\left(d\right)=\sum_{s\in N\left(d\right)}D_{d}\left(s\right)$$ (8)

In general, the similarity between the two diseases is higher if the nodes sharing in their DAGs are higher. Therefore, we compute the semantic similarity of diseases $d_{i}$ and $d_{j}$ using the following formula:

$$SV\left(d_{i},d_{j}\right)=\frac{\sum_{s\in N\left(d_{i}\right)\cap N\left(d_{j}\right)}\left(D_{d_{i}}\left(s\right)+D_{d_{j}}\left(s\right)\right)}{DV\left(d_{i}\right)+DV\left(d_{j}\right)}$$ (9)

### Long non-coding RNA Functional Similarity

We computed the functional similarities of lncRNAs according to the LNCSIM model (Chen et al., 2015). Let $D\left(l_{i}\right)$ and $D\left(l_{j}\right)$ denoted the corresponding disease sets of lncRNA $l_{i}$ and lncRNA $l_{j}$ , and the similarity between disease d and the disease set of lncRNA $l_{j}$ ( $D\left(l_{j}\right)$ ) is given by

$$S\left(d,D\left(l_{j}\right)\right)=\underset{d'\in D\left(l_{j}\right)}{\max}\left(SV\left(d,d'\right)\right)$$ (10)

In view of the hypothesis that functionally similar lncRNAs are usually related with similar diseases, the functional similarity between lncRNAs $l_{i}$ and $l_{j}$ is computed as follows:

$$FL\left(l_{i},l_{j}\right)=\frac{\sum_{d\in D\left(l_{i}\right)}S\left(d,D\left(l_{j}\right)\right)+\sum_{d\in D\left(l_{j}\right)}S\left(d,D\left(l_{i}\right)\right)}{\left|D\left(l_{i}\right)\right|+\left|D\left(l_{j}\right)\right|}$$ (11)

where $\left|D\left(l_{i}\right)\right|$ denotes the number of elements in $D\left(l_{i}\right)$ .

### Network Consistency Projection and Bi-Random Walk

**FIGURE 1:**
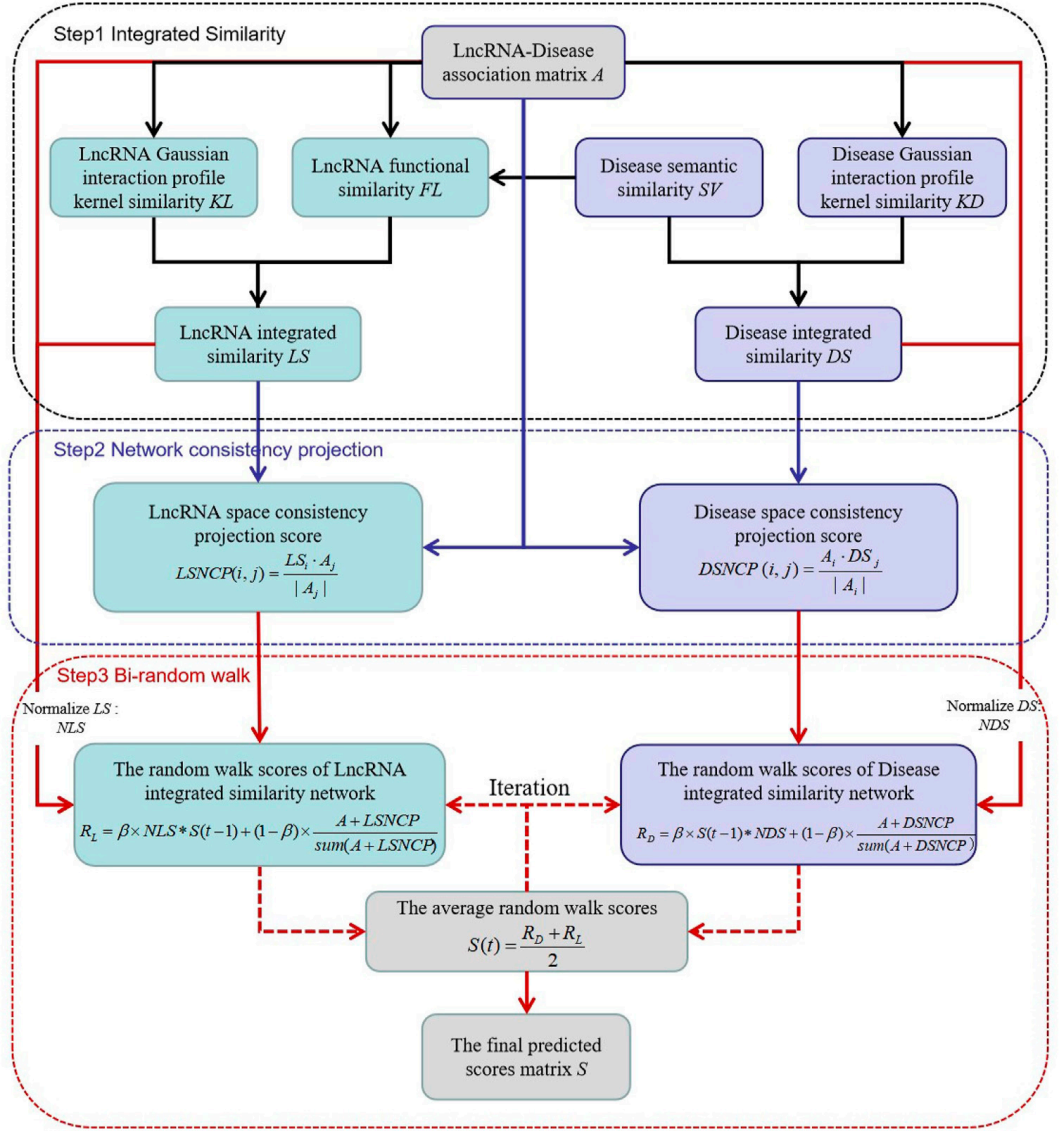
Flow chart of NCP-BiRW.

We constructed a novel model NCP-BiRW involving network consistent projection (Xie et al., 2019) and bi-random walk (Hu et al., 2019) to forecast hidden lncRNA-disease interactions. We divided the model implementation process into three steps. Figure 1 shows the flowchart of the algorithm.

Step 1 construction of integrated similarity networks for lncRNAs and diseases

The integrated technique was adopted to obtain more similarity information. On the basis of the lncRNA GIP kernel similarity matrix (*KL*) and the lncRNA functional similarity matrix (*FL*), the integrated similarity between lncRNAs $l_{i}$ and $l_{j}$ is as follows:

$$LS\left(l_{i},l_{j}\right)=\{\begin{array}{cc} \frac{KL\left(l_{i},l_{j}\right)+FL\left(l_{i},l_{j}\right)}{2} & if\;FL\left(l_{i},l_{j}\right)\neq 0\\ KL\left(l_{i},l_{j}\right) & otherwise \end{array}$$ (12)

Similarly, based on the disease semantic similarity matrix (*SV*) and the disease GIP kernel similarity matrix (*KD*), the integrated similarity between diseases $d_{i}$ and $d_{j}$ is as follows:

$$DS\left(d_{i},d_{j}\right)=\{\begin{array}{cc} \frac{KD\left(d_{i},d_{j}\right)+SV\left(d_{i},d_{j}\right)}{2} & if\;SV\left(d_{i},d_{j}\right)\neq 0\\ KD\left(d_{i},d_{j}\right) & otherwise \end{array}$$ (13)

Step 2 network consistency projection for lncRNA and disease spaces

We constructed a heterogeneous network consisting of the above integrated similarity networks and lncRNA-disease association network. The network consistency projection method was utilized to obtain more network topological information (Yin et al., 2020). Network consistency projection can be divided into lncRNA network consistency projection and disease network consistency projection (Li et al., 2019; Xie et al., 2019).

The lncRNA network consistency projection fractions can be formulated as follows:

$$LSNCP\left(i,j\right)=\frac{LS_{i}\cdot A_{j}}{\left|A_{j}\right|}$$ (14)

where $LS_{i}$ is the *i*-th row of the lncRNA integrated similarity matrix (*LS*). $A_{j}$ is the *j*-th column of the association matrix A , $A_{j}$ represents the relevance between disease $d_{j}$ and all lncRNAs, $\left|A_{j}\right|$ is the norm of $A_{j}$ , and LSNCP(i,j) is the projection fraction of $LS_{i}$ on $A_{j}$ . In particular, if the angle between $LS_{i}$ and $A_{j}$ is smaller, the score LSNCP(i,j) is higher (Bao et al., 2017).

Similarly, the formula of the disease network consistency projection fractions is as follows:

$$DSNCP\left(i,j\right)=\frac{A_{i}\cdot DS_{j}}{\left|A_{i}\right|}$$ (15)

where $DS_{j}$ is the *j*-th column of the disease integrated similarity matrix (*DS*), $A_{i}$ is the *i*-th row of A (representing the relevance between lncRNA $l_{i}$ and all diseases), and DSNCP(i,j) is the projection fraction of $DS_{j}$ on $A_{i}$ .

Step 3 bi-random walk in the integrated similarity networks of lncRNAs and diseases

First, the integrated similarity networks, *LS* and *DS* were normalized such that all the similarity values were between 0 and 1 (Hu et al., 2019). The formula of the normalized similarity of lncRNAs is as follows:

$$NLS\left(i,j\right)=\frac{LS\left(i,j\right)}{\sqrt{\sum_{i}LS\left(i,j\right)\sum_{j}LS\left(i,j\right)}}$$ (16)

Similarly, the normalization of the disease similarity is as follows:

$$NDS\left(i,j\right)=\frac{DS\left(i,j\right)}{\sqrt{\sum_{i}DS\left(i,j\right)\sum_{j}DS\left(i,j\right)}}$$ (17)

The association matrix A should also be normalized, as follows:

$$S\left(0\right)=\frac{A}{sum\left(A\right)}$$ (18)

Then, we carried out the random walk method for both the lncRNA similarity network and the disease similarity network, called bi-random walk, a global process (Zhang et al., 2018). *r*_1_ and *r*_2_ are designated as the maximum number of iterations in the lncRNA and disease similarity networks, respectively. If *r*_1_ > *r*_2_, the lncRNA similarity is considered more important in the predicted process (Hu et al., 2019). On the basis of the results of the network consistency projection, the iteration processes are as follows:

$$R_{L}=\beta \times NLS*S\left(t-1\right)+\left(1-\beta \right)\times \frac{A+LSNCP}{sum\left(A+LSNCP\right)}$$ (19)

$$R_{D}=\beta \times S\left(t-1\right)*NDS+\left(1-\beta \right)\times \frac{A+DSNCP}{sum\left(A+DSNCP\right)}$$ (20)

$$S\left(t\right)=\frac{R_{L}+R_{D}}{2}$$ (21)

where $R_{L}$ and $R_{D}$ denote the random walk scores in the lncRNA similarity network and the disease similarity network, respectively. *β* is the decay factor that controls the proportion of primitive information, *NLS* and *NDS* denote the lncRNA and disease normalized integrated similarity matrices, respectively. S(0) is the initial probability matrix of *A*, and the iterative function S(t) denotes the average value of $R_{L}$ and $R_{D}$ in step *t*. When $t=\max\left\{r_{1},r_{2}\right\}$ , the algorithm ends, and we obtain the final S(t) (denoted as *S*), which contains all the predictive scores of lncRNA-disease pairs.

## Results

### Performance Evaluation

We used *k*-fold CV to evaluate the model performance. In *k*-fold CV, known lncRNA-disease pairs are divided into *k* subparts, with *k*-1 parts as the training set and the remaining part as the testing set. Here, we chose *k* = 5 (5-fold CV) and *k* = 10 (10-fold CV). All unknown associations were regarded as candidate samples. The predicted score of each lncRNA-disease pair was obtained using NCP-BiRW. The predicted scores of the test and candidate samples were sorted together. The receiver operating characteristic (ROC) curve was drawn according to the false positive rate (FPR) and the true positive rate (TPR) under different thresholds. The area under the ROC curve (AUC) was employed as a metric to assess the overall performance of our method. For AUC ∈[0,1] , when the value is closer to 1, the model performs better.

### Effects of Parameters

**FIGURE 2:**
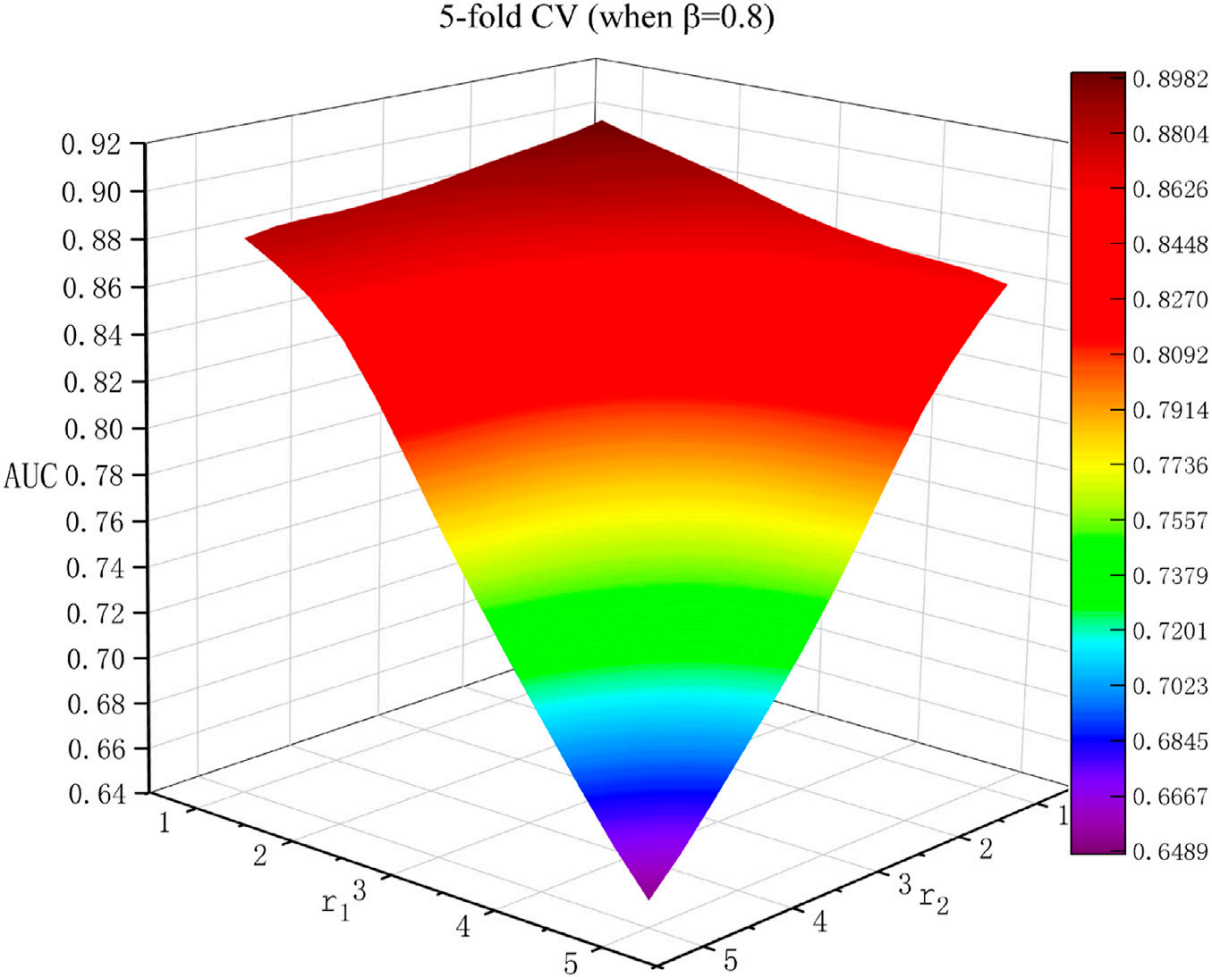
Results for *r*_1_ and *r*_2_ when β *=* 0.8 in 5-fold CV.

In this research, there were three parameters: β , *r*_1_ and *r*_2_. β denotes the decay factor in bi-random walk, and its value ranges from 0 to 1. To test the performance of the model, we increased β from 0.1 to 0.9 in steps of 0.1. The maximum number of iterations in the lncRNA and disease similarity networks (*r*_1_ and *r*_2_, respectively) was from 1 to 5, evaluated with a step size of 1. The grid search algorithm was used to determine the proper values of these parameters. By experimental comparison, the best parameter values were β *=* 0.8 and *r*_1_ = *r*_2_ = 1 in the 5-fold CV framework, whereas in 10-fold CV framework, the optimal values were β *=* 0.7 and *r*_1_ = *r*_2_ = 1. The experimental results of the grid search are listed in Supplementary Table S1. In the 10-fold CV framework, when β *=* 0.7 and *r*_1_ = *r*_2_ = 1, the AUC value was close to the best AUC value. Finally, we set β *=* 0.8 and *r*_1_ = *r*_2_ = 1 in the proposed model. Figure 2 shows the experimental effects of different *r*_1_ and *r*_2_ values when β *=* 0.8 in the 5-fold CV framework. The optimal parameters corresponding to the best AUC were *r*_1_ = *r*_2_ = 1.

### Comparison With Other Methods

**FIGURE 3:**
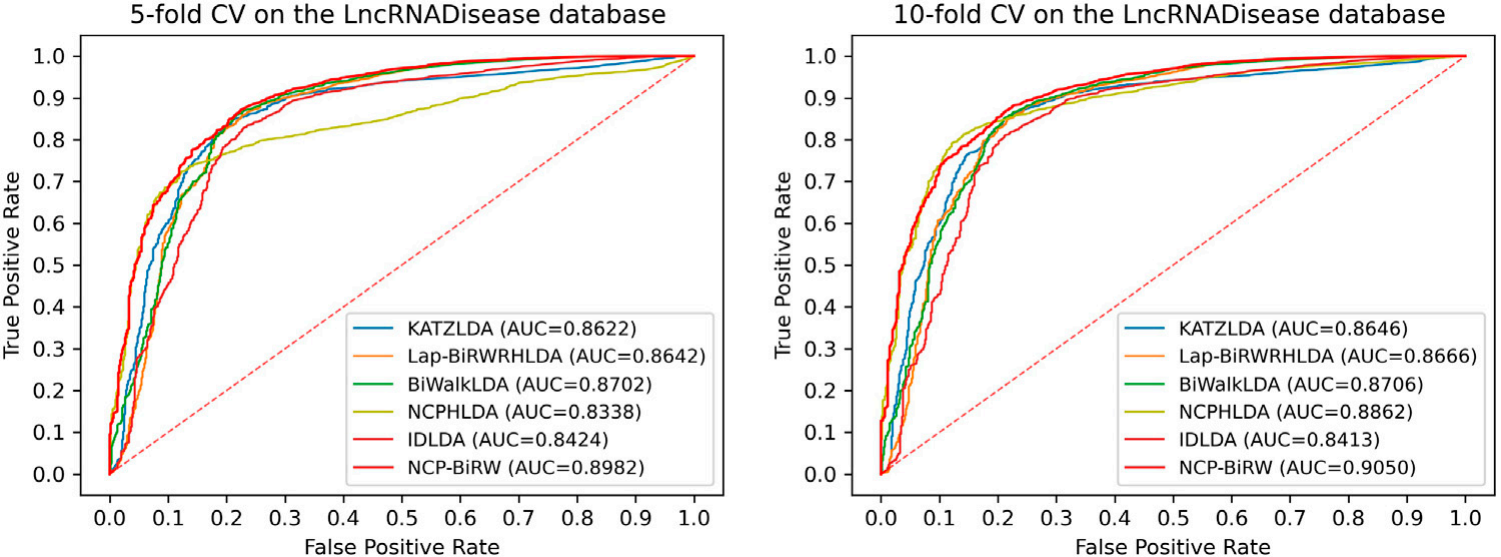
ROCs and AUCs of the six methods using the LncRNADisease database.

**FIGURE 4:**
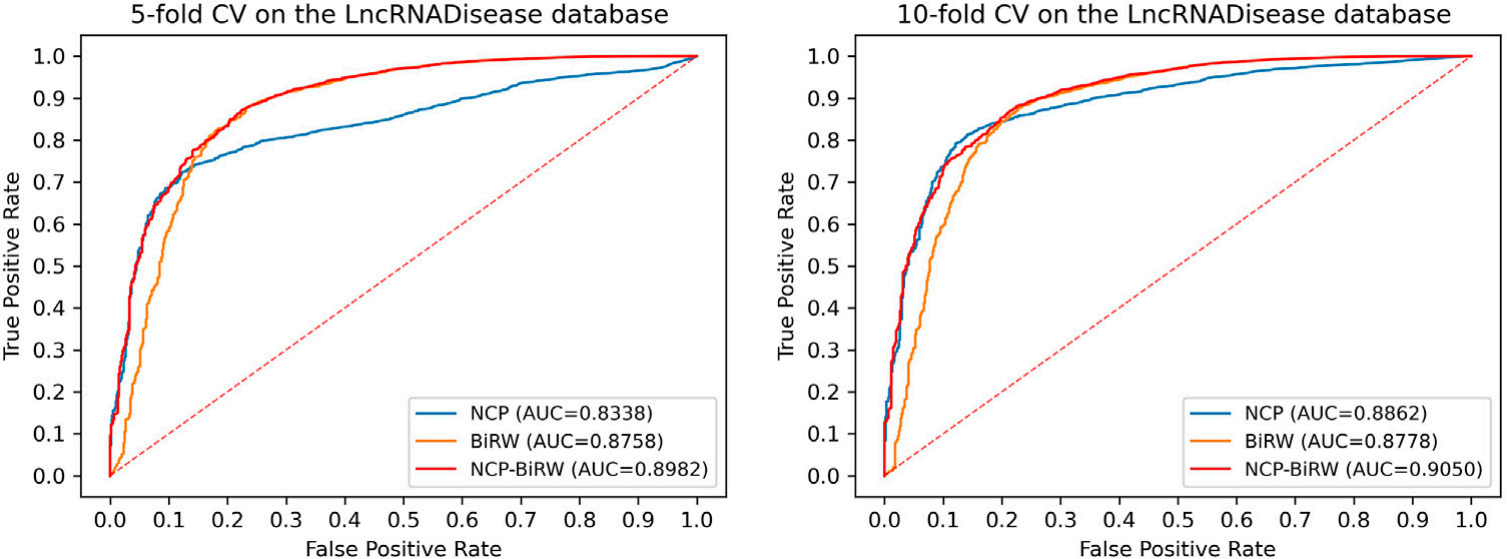
Comparisons of NCP, BiRW, and NCP-BiRW using the LncRNADisease database.

In order to prove the excellent model performance, we compared NCP-BiRW with five other popular algorithms: KATZLDA (Chen, 2015), Lap-BiRWRHLDA (Wen et al., 2018), BiWalkLDA (Hu et al., 2019), NCPHLDA (Xie et al., 2019), and IDLDA (Wang and Yan, 2019). We chose the parameter values for each model in the original reference. First, we conducted 5-fold CV, as shown in Figure 3, and the AUC of NCP-BiRW was 0.8982, which was better than the AUC values of the other five methods (KATZLDA: 0.8622, Lap-BiRWRHLDA: 0.8642, BiWalkLDA: 0.8702, NCPHLDA: 0.8338, and IDLDA: 0.8424). Then, we conducted 10-fold CV, and the AUC of NCP-BiRW was 0.9050 (Figure 3), which had the best performance (KATZLDA: 0.8646, Lap-BiRWRHLDA: 0.8666, BiWalkLDA: 0.8706, NCPHLDA: 0.8862, and IDLDA: 0.8413). In addition, we considered the following two models: 1) NCP, i.e., NCP-BiRW without bi-random walk; and 2) BiRW, i.e., NCP-BiRW without network consistent projection. Then, we compared the two models with NCP-BiRW, as shown in Figure 4. The results showed that our hybrid method was better than every single method. In summary, NCP-BiRW achieved the best performance for predicting lncRNA-disease interactions using the dataset from the LncRNADisease database.

### Robustness of Evaluation Using Another Dataset

**FIGURE 5:**
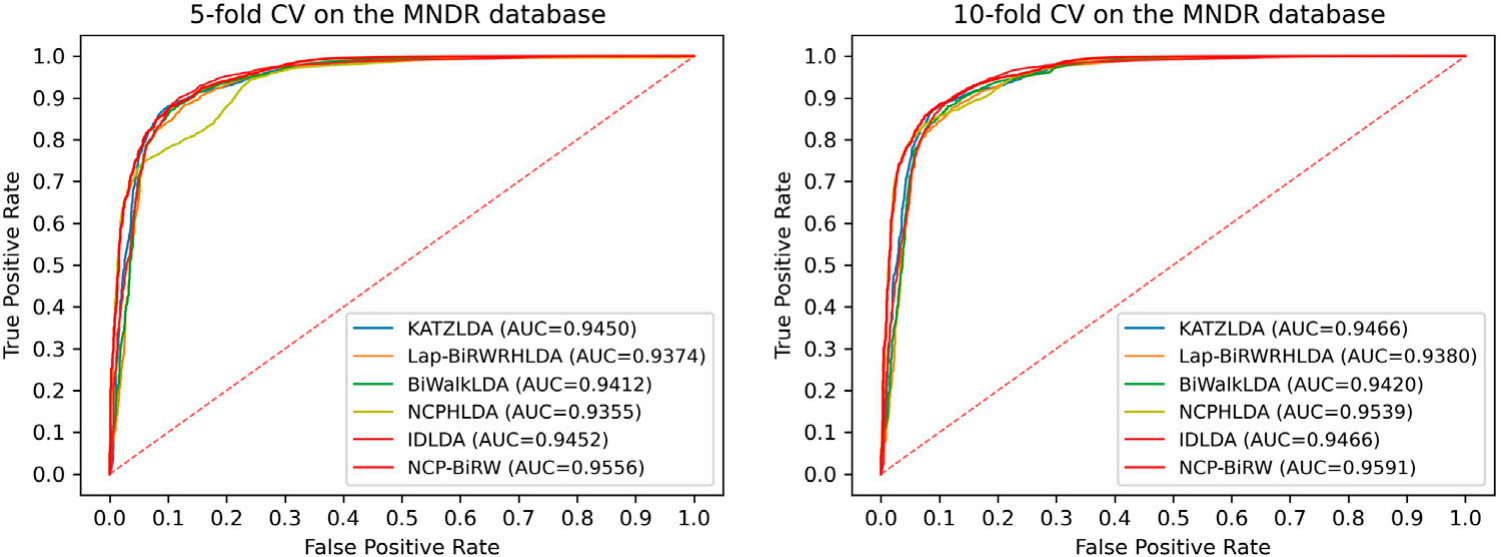
ROCs and AUCs of the six methods using the MNDR database.

We then applied NCP-BiRW to another dataset to determine whether our method could still achieve outstanding performance. We chose the Mammalian ncRNA-Disease Repository (MNDR) database (Cui et al., 2018), from which the known lncRNA-disease interactions were downloaded. After data cleaning, 1,680 known interactions between 190 diseases and 89 lncRNAs were selected (Fan et al., 2020). We performed the same experiment as above, and Figure 5 shows the final computational results. In 5-fold CV, the AUC of NCP-BiRW was 0.9556, which was better than those of KATZLDA (0.9450), Lap-BiRWRHLDA (0.9374), BiWalkLDA (0.9412), NCPHLDA (0.9355), and IDLDA (0.9452). In 10-fold CV, NCP-BiRW also performed the best. The AUCs of KATZLDA, Lap-BiRWRHLDA, BiWalkLDA, NCPHLDA, IDLDA and NCP-BiRW were 0.9466, 0.9380, 0.9420, 0.9539, 0.9466 and 0.9591, respectively. The excellent performance of NCP-BiRW using the MNDR database demonstrated the robustness of our model.

### Case Studies

Next, we chose atherosclerosis (AS) and leukemia as model diseases, and conducted case studies using these two diseases to further confirm the predictive effects of NCP-BiRW. The top 10 candidate lncRNAs predicted by our method for the two diseases are listed in Tables 1, 2. Eventually, lncRNAs in the tables were verified using the MNDR database (Ning et al., 2021) and the Lnc2Cancer database (Gao et al., 2021) (http://bio-bigdata.hrbmu.edu.cn/lnc2cancer).

**TABLE1:** Top ten lncRNAs for atherosclerosis.

Rank	LncRNA	Evidence
1	*MALAT1*	MNDR
2	*MEG3*	MNDR
3	*HOTAIR*	MNDR
4	*PVT1*	Unknown
5	*GAS5*	MNDR
6	*UCA1*	MNDR
7	*TUG1*	MNDR
8	*BCYRN1*	Unknown
9	*XIST*	MNDR
10	*SPRY4-IT1*	Unknown

**TABLE 2:** Top ten lncRNAs for leukemia.

Rank	LncRNA	Evidence
1	*H19*	Lnc2Cancer
2	*MEG3*	Lnc2Cancer
3	*MALAT1*	Lnc2Cancer
4	*HOTAIR*	MNDR, Lnc2Cancer
5	*PVT1*	MNDR, Lnc2Cancer
6	*GAS5*	MNDR, Lnc2Cancer
7	*UCA1*	Lnc2Cancer
8	*TUG1*	Lnc2Cancer
9	*MIAT*	Lnc2Cancer
10	*XIST*	MNDR, Lnc2Cancer

AS is a chronic inflammatory disease characterized by lipid-rich plaques in the artery wall (Vigario et al., 2020). AS is the primary cause of most cardiovascular diseases, including acute myocardial infarction and stroke (Li et al., 2020). Many lncRNAs have been shown to function in AS, the central underlying pathology of cardiovascular diseases (Josefs and Boon, 2020). In this study, we next predicted the top 10 lncRNAs associated with AS (Table 1). Seven of these top 10 lncRNAs were verified using the MNDR database. For example, *MALAT1* (ranked first) inhibits AS through *miR-155* and *SOCS1*. Specifically, *MALAT1* inhibits the release of inflammatory cytokines and blocks apoptosis by sponging *miR-155* and enhancing *SOCS1* expression to suppress the Janus kinase/signal transducer and activator of the transcription pathway (Li et al., 2018). Additionally, *MEG3* (ranked second), an endothelial-enriched lncRNA, acts as a competing endogenous RNA against *miR-223*, which may explain the anti-AS functions of melatonin (Zhang et al., 2018). *HOTAIR* (ranked third), is related to the progression of various cancers; however, its functions in AS are still unclear. Notably, *HOTAIR* has been shown to control AS progression by sponging *miR-330-5p* in THP-1 cells (Liu et al., 2019).

Leukemia, a type of blood or bone marrow cancer, involves excessive production of white cells (Luo et al., 2015). There are four main types of leukemia: acute lymphocytic leukemia, acute myeloid leukemia (AML), chronic lymphocytic leukemia, and chronic myeloid leukemia (CML) (Siegel et al., 2021). In 2020, over 31,000 people died of leukemia worldwide (Siegel et al., 2021). Recent studies have demonstrated the relationships among lncRNAs and the pathophysiology of leukemia (Gao et al., 2020). The top 10 predicted leukemia-related lncRNAs are listed in Table 2. All 10 were validated using the Lnc2Cancer database and the MNDR database. *MALAT1* (ranked third) promotes the survival of CML cells, stimulates the cell cycle and imatinib resistance by sponging *miR-328*, highlighting the vital roles of *MALAT1* as a microRNA sponge in CML and supporting the application of lncRNA-targeted therapies in the treatment of CML (Wen et al., 2018). Additionally, *TUG1* (ranked eighth) promotes the progression of AML through the *miR-370-3p*/mitogen-activated protein kinase 1 (MAPK1)/extracellular signal-regulated kinase (ERK) signaling pathway. The MAPK1/ERK signaling pathway inhibits the epithelial-mesenchymal transition and thus blocks the migration and invasion of AML cells (Li et al., 2019). Studies have shown that *MIAT* (ranked ninth) is highly expressed in various solid tumors in humans and promotes AML progression by negatively regulating *miR-495*, which may be a promising therapeutic target in patients with AML (Wang et al., 2019).

## Discussion

According to a substantial body of evidence, lncRNAs are critical for disease research. Identification of hidden lncRNA-disease pairs may provide insights into the pathological mechanisms of diseases, disease prevention, diagnosis, and treatment. Experimental techniques have been used to identify unknown lncRNA-disease interactions; however, these approaches are slow and costly. Therefore, computing methods have been developed as alternative approaches. Here, we constructed a new algorithm, NCP-BiRW, based on network consistency projection and bi-random walk. First, we integrated two similarity networks, i.e., one for diseases combining disease GIP kernel similarity and disease semantic similarity, and the other for lncRNAs combining lncRNA functional similarity and lncRNA GIP kernel similarity. Then, we used NCP-BiRW to forecast lncRNA-disease interactions on the LncRNADisease database. To validate its superiority, NCP-BiRW was compared with five classical models: KATZLDA, Lap-BiRWRHLDA, BiWalkLDA, NCPHLDA, and IDLDA based on 5- and 10-fold CV frameworks. The AUCs of NCP-BiRW were 0.8982 and 0.9050 for the two frameworks, respectively. To further test the stability of NCP-BiRW, we applied six methods on the MNDR database. After the same experimental process, the performance of NCP-BiRW was found to be optimal. Furthermore, case studies on AS and leukemia were used to validate the predictive performance of our algorithm in practice, and the prediction accuracy of the top 10 lncRNAs in AS and leukemia were 70% and 100%, respectively.

The reasons for the outstanding performance of our model are as follows. First, a considerable amount of biological information about lncRNAs and diseases was applied. Indeed, we used disease semantic similarity, GIP kernel similarity, and lncRNA functional similarity to construct similarity networks. Second, we did not use negative samples. Third, for making full use of network topological information, network consistency projection was applied. Moreover, no parameters were necessary for this step, so the computational efficiency was improved. Finally, the model added the results of network consistency projection into the bi-random walk, so more network topological information was added to the initial association matrix in the computing process of the bi-random walk method. By conducting random walks on two similarity networks, the similarity of lncRNAs and diseases are used reasonably and fully. Based on the above, the performance of the algorithm has been improved. In the future, our model can be used for other association predictions, such as miRNA-disease, gene-disease, drug-disease associations.

Despite these advantages, there are still some limitations of the NCP-BiRW framework. First, the proportion of known lncRNA-disease interactions in the LncRNADisease database is only 5.4%, and the original association matrix is thus very sparse; this could influence various calculations, including GIP kernel similarity, network consistency projection, and bi-random walk. Second, in this study, we only considered two factors: lncRNAs and diseases, and more biological information on different factors (such as genes, protein, and other types of RNAs) may provide more evidence for the prediction of lncRNA-disease interactions. Therefore, more valuable biological information is necessary for the future. Finally, NCP-BiRW is a network-based method. With the emergence of new methods in different fields, developing more algorithms for integration of various fields is essential. In our future studies, we will plan to apply multiple types of data with more biological information to association prediction models in order to yield more accurate predictive effects.

## Data Availability

Publicly available datasets were analyzed in this study. This data can be found here: https://github.com/CDMB-lab/IDSSIM.
