# Supplementary material for: NCP-BiRW: A Hybrid Approach for Predicting Long Noncoding RNA-Disease Associations by Network Consistency Projection and Bi-Random Walk
Source: Front Genet. 2022 Apr 13;13:862272. doi: 10.3389/fgene.2022.862272 (PMC9043107; doi:10.3389/fgene.2022.862272)
Supplement: Supplementary file 1 [file DataSheet1.docx]

Supplementary Material

# Supplementary Tables

- 1. **Supplementary file 1 (Five-fold CV)**

| *β=* 0.1 | | *r*_2_ | | | | |
| --- | --- | --- | --- | --- | --- | --- |
|  |  | 1 | 2 | 3 | 4 | 5 |
| *r*_1_ | 1 | **0.7810** | 0.7756 | 0.7754 | 0.7754 | 0.7754 |
|  | 2 | 0.7683 | 0.7606 | 0.7597 | 0.7597 | 0.7597 |
|  | 3 | 0.7677 | 0.7592 | 0.7583 | 0.7583 | 0.7583 |
|  | 4 | 0.7676 | 0.7592 | 0.7582 | 0.7582 | 0.7582 |
|  | 5 | 0.7676 | 0.7592 | 0.7582 | 0.7582 | 0.7582 |

| *β=* 0.2 | | *r*_2_ | | | | |
| --- | --- | --- | --- | --- | --- | --- |
|  |  | 1 | 2 | 3 | 4 | 5 |
| *r*_1_ | 1 | **0.8023** | 0.7892 | 0.7884 | 0.7883 | 0.7883 |
|  | 2 | 0.7766 | 0.7648 | 0.7620 | 0.7618 | 0.7618 |
|  | 3 | 0.7743 | 0.7601 | 0.7568 | 0.7563 | 0.7563 |
|  | 4 | 0.7741 | 0.7597 | 0.7560 | 0.7555 | 0.7554 |
|  | 5 | 0.7741 | 0.7596 | 0.7560 | 0.7554 | 0.7553 |

| *β=* 0.3 | | *r*_2_ | | | | |
| --- | --- | --- | --- | --- | --- | --- |
|  |  | 1 | 2 | 3 | 4 | 5 |
| *r*_1_ | 1 | **0.8303** | 0.8095 | 0.8070 | 0.8067 | 0.8067 |
|  | 2 | 0.7873 | 0.7715 | 0.7666 | 0.7660 | 0.7660 |
|  | 3 | 0.7822 | 0.7626 | 0.7558 | 0.7541 | 0.7539 |
|  | 4 | 0.7815 | 0.7613 | 0.7532 | 0.7513 | 0.7509 |
|  | 5 | 0.7814 | 0.7611 | 0.7528 | 0.7507 | 0.7503 |

| *β=* 0.4 | | *r*_2_ | | | | |
| --- | --- | --- | --- | --- | --- | --- |
|  |  | 1 | 2 | 3 | 4 | 5 |
| *r*_1_ | 1 | **0.8557** | 0.8330 | 0.8285 | 0.8278 | 0.8276 |
|  | 2 | 0.8048 | 0.7802 | 0.7729 | 0.7720 | 0.7718 |
|  | 3 | 0.7947 | 0.7671 | 0.7564 | 0.7525 | 0.7519 |
|  | 4 | 0.7929 | 0.7643 | 0.7501 | 0.7455 | 0.7439 |
|  | 5 | 0.7925 | 0.7638 | 0.7490 | 0.7432 | 0.7416 |

| *β=* 0.5 | | *r*_2_ | | | | |
| --- | --- | --- | --- | --- | --- | --- |
|  |  | 1 | 2 | 3 | 4 | 5 |
| *r*_1_ | 1 | **0.8745** | 0.8544 | 0.8483 | 0.8469 | 0.8465 |
|  | 2 | 0.8289 | 0.7935 | 0.7818 | 0.7801 | 0.7798 |
|  | 3 | 0.8141 | 0.7737 | 0.7596 | 0.7527 | 0.7513 |
|  | 4 | 0.8106 | 0.7690 | 0.7482 | 0.7384 | 0.7342 |
|  | 5 | 0.8097 | 0.7678 | 0.7450 | 0.7322 | 0.7274 |

| *β=* 0.6 | | *r*_2_ | | | | |
| --- | --- | --- | --- | --- | --- | --- |
|  |  | 1 | 2 | 3 | 4 | 5 |
| *r*_1_ | 1 | **0.8873** | 0.8720 | 0.8650 | 0.8626 | 0.8618 |
|  | 2 | 0.8537 | 0.8190 | 0.7981 | 0.7946 | 0.7937 |
|  | 3 | 0.8358 | 0.7848 | 0.7663 | 0.7560 | 0.7535 |
|  | 4 | 0.8304 | 0.7766 | 0.7489 | 0.7321 | 0.7223 |
|  | 5 | 0.8288 | 0.7742 | 0.7427 | 0.7175 | 0.7049 |

| *β=* 0.7 | | *r*_2_ | | | | |
| --- | --- | --- | --- | --- | --- | --- |
|  |  | 1 | 2 | 3 | 4 | 5 |
| *r*_1_ | 1 | **0.8951** | 0.8843 | 0.8777 | 0.8750 | 0.8740 |
|  | 2 | 0.8739 | 0.8529 | 0.8276 | 0.8210 | 0.8190 |
|  | 3 | 0.8570 | 0.8093 | 0.7775 | 0.7644 | 0.7605 |
|  | 4 | 0.8500 | 0.7937 | 0.7538 | 0.7302 | 0.7119 |
|  | 5 | 0.8474 | 0.7885 | 0.7438 | 0.7030 | 0.6743 |

| *β=* 0.8 | | *r*_2_ | | | | |
| --- | --- | --- | --- | --- | --- | --- |
|  |  | 1 | 2 | 3 | 4 | 5 |
| *r*_1_ | 1 | **0.8982** | 0.8925 | 0.8859 | 0.8821 | 0.8804 |
|  | 2 | 0.8877 | 0.8799 | 0.8630 | 0.8547 | 0.8513 |
|  | 3 | 0.8747 | 0.8500 | 0.8081 | 0.7839 | 0.7777 |
|  | 4 | 0.8670 | 0.8293 | 0.7674 | 0.7378 | 0.7116 |
|  | 5 | 0.8634 | 0.8205 | 0.7527 | 0.6991 | 0.6489 |

| *β=* 0.9 | | *r*_2_ | | | | |
| --- | --- | --- | --- | --- | --- | --- |
|  |  | 1 | 2 | 3 | 4 | 5 |
| *r*_1_ | 1 | **0.8949** | 0.8923 | 0.8877 | 0.8835 | 0.8808 |
|  | 2 | 0.8934 | 0.8909 | 0.8857 | 0.8813 | 0.8785 |
|  | 3 | 0.8844 | 0.8774 | 0.8593 | 0.8367 | 0.8247 |
|  | 4 | 0.8782 | 0.8642 | 0.8218 | 0.7741 | 0.7380 |
|  | 5 | 0.8744 | 0.8566 | 0.7977 | 0.7196 | 0.6578 |

**1.2 Supplementary file 2 (Ten-fold CV)**

| *β=* 0.1 | | *r*_2_ | | | | |
| --- | --- | --- | --- | --- | --- | --- |
|  |  | 1 | 2 | 3 | 4 | 5 |
| *r*_1_ | 1 | **0.8455** | 0.8424 | 0.8423 | 0.8423 | 0.8423 |
|  | 2 | 0.8373 | 0.8326 | 0.8322 | 0.8322 | 0.8322 |
|  | 3 | 0.8368 | 0.8319 | 0.8314 | 0.8314 | 0.8314 |
|  | 4 | 0.8368 | 0.8318 | 0.8314 | 0.8313 | 0.8313 |
|  | 5 | 0.8368 | 0.8318 | 0.8314 | 0.8313 | 0.8313 |

| *β=* 0.2 | | *r*_2_ | | | | |
| --- | --- | --- | --- | --- | --- | --- |
|  |  | 1 | 2 | 3 | 4 | 5 |
| *r*_1_ | 1 | **0.8568** | 0.8512 | 0.8508 | 0.8508 | 0.8508 |
|  | 2 | 0.8428 | 0.8361 | 0.8346 | 0.8345 | 0.8345 |
|  | 3 | 0.8414 | 0.8334 | 0.8317 | 0.8315 | 0.8315 |
|  | 4 | 0.8412 | 0.8332 | 0.8314 | 0.8312 | 0.8311 |
|  | 5 | 0.8412 | 0.8332 | 0.8313 | 0.8311 | 0.8311 |

| *β=* 0.3 | | *r*_2_ | | | | |
| --- | --- | --- | --- | --- | --- | --- |
|  |  | 1 | 2 | 3 | 4 | 5 |
| *r*_1_ | 1 | **0.8708** | 0.8614 | 0.8605 | 0.8604 | 0.8604 |
|  | 2 | 0.8487 | 0.8407 | 0.8380 | 0.8378 | 0.8378 |
|  | 3 | 0.8458 | 0.8356 | 0.8322 | 0.8315 | 0.8314 |
|  | 4 | 0.8454 | 0.8348 | 0.8309 | 0.8301 | 0.8300 |
|  | 5 | 0.8453 | 0.8348 | 0.8307 | 0.8299 | 0.8297 |

| *β=* 0.4 | | *r*_2_ | | | | |
| --- | --- | --- | --- | --- | --- | --- |
|  |  | 1 | 2 | 3 | 4 | 5 |
| *r*_1_ | 1 | **0.8843** | 0.8736 | 0.8717 | 0.8714 | 0.8714 |
|  | 2 | 0.8570 | 0.8461 | 0.8424 | 0.8420 | 0.8419 |
|  | 3 | 0.8515 | 0.8384 | 0.8333 | 0.8316 | 0.8314 |
|  | 4 | 0.8505 | 0.8370 | 0.8304 | 0.8285 | 0.8279 |
|  | 5 | 0.8503 | 0.8367 | 0.8298 | 0.8276 | 0.8270 |

| *β=* 0.5 | | *r*_2_ | | | | |
| --- | --- | --- | --- | --- | --- | --- |
|  |  | 1 | 2 | 3 | 4 | 5 |
| *r*_1_ | 1 | **0.8949** | 0.8854 | 0.8828 | 0.8822 | 0.8821 |
|  | 2 | 0.8691 | 0.8533 | 0.8477 | 0.8470 | 0.8469 |
|  | 3 | 0.8603 | 0.8422 | 0.8353 | 0.8325 | 0.8320 |
|  | 4 | 0.8582 | 0.8396 | 0.8299 | 0.8263 | 0.8249 |
|  | 5 | 0.8577 | 0.8389 | 0.8285 | 0.8239 | 0.8222 |

| *β=* 0.6 | | *r*_2_ | | | | |
| --- | --- | --- | --- | --- | --- | --- |
|  |  | 1 | 2 | 3 | 4 | 5 |
| *r*_1_ | 1 | **0.9018** | 0.8947 | 0.8914 | 0.8904 | 0.8900 |
|  | 2 | 0.8821 | 0.8654 | 0.8561 | 0.8547 | 0.8543 |
|  | 3 | 0.8714 | 0.8474 | 0.8384 | 0.8342 | 0.8334 |
|  | 4 | 0.8679 | 0.8429 | 0.8301 | 0.8239 | 0.8207 |
|  | 5 | 0.8669 | 0.8417 | 0.8272 | 0.8184 | 0.8145 |

| *β=* 0.7 | | *r*_2_ | | | | |
| --- | --- | --- | --- | --- | --- | --- |
|  |  | 1 | 2 | 3 | 4 | 5 |
| *r*_1_ | 1 | **0.9051** | 0.9009 | 0.8973 | 0.8960 | 0.8954 |
|  | 2 | 0.8927 | 0.8826 | 0.8701 | 0.8673 | 0.8666 |
|  | 3 | 0.8821 | 0.8583 | 0.8434 | 0.8375 | 0.8362 |
|  | 4 | 0.8776 | 0.8499 | 0.8312 | 0.8220 | 0.8159 |
|  | 5 | 0.8760 | 0.8470 | 0.8262 | 0.8111 | 0.8024 |

| *β=* 0.8 | | *r*_2_ | | | | |
| --- | --- | --- | --- | --- | --- | --- |
|  |  | 1 | 2 | 3 | 4 | 5 |
| *r*_1_ | 1 | **0.9050** | 0.9027 | 0.8993 | 0.8971 | 0.8962 |
|  | 2 | 0.8992 | 0.8961 | 0.8880 | 0.8842 | 0.8827 |
|  | 3 | 0.8895 | 0.8771 | 0.8561 | 0.8451 | 0.8428 |
|  | 4 | 0.8840 | 0.8648 | 0.8341 | 0.8210 | 0.8112 |
|  | 5 | 0.8814 | 0.8596 | 0.8253 | 0.8022 | 0.7848 |

| *β=* 0.9 | | *r*_2_ | | | | |
| --- | --- | --- | --- | --- | --- | --- |
|  |  | 1 | 2 | 3 | 4 | 5 |
| *r*_1_ | 1 | **0.9000** | 0.8985 | 0.8949 | 0.8921 | 0.8904 |
|  | 2 | 0.8990 | 0.8982 | 0.8955 | 0.8935 | 0.8921 |
|  | 3 | 0.8919 | 0.8879 | 0.8774 | 0.8659 | 0.8602 |
|  | 4 | 0.8863 | 0.8775 | 0.8518 | 0.8273 | 0.8110 |
|  | 5 | 0.8828 | 0.8711 | 0.8360 | 0.7951 | 0.7645 |
